# 

**Published:** 1921-09

**Authors:** 


*The following donations have been received since the publication of the List in June.*

*October* 1*st*, 1921.

| | |
|---|---|
| Dr. F. W. E. Burnham (1) .. .. .. | 1 volume. |
| Dr. Carey Coombs (2) .. .. .. .. | 1 ,, |
| The late Dr. Fortescue-Brickdale (3) .. .. | 293 volumes. |
| Dr. J. R. Kay-Mouat (4) .. .. .. .. | 4 ,, |
| Dr. P. W. McCrea (5) .. .. .. .. | 4 ,, |
| Dr. P. Watson-Williams (6) .. .. .. | 6 ,, |
| Dr. J. O. Symes (7) .. .. .. .. | 1 volume. |

### THE ONE HUNDRED AND FOURTH LIST OF BOOKS.

The figures in brackets refer to the figures after the names of the donors, and show by whom the volumes were presented. The books to which no such figures are attached have either been bought from the Library Fund or received through the *Journal.*

| | | | |
|---|---|---|---|
| **Abrahams and A. C. Morson, A.** | *A Guide to Urinary Diseases* | | 1921 |
| **Alexander, J.** | *The Cure of Self-consciousness* | (4) | 1915 |
| **Annesley, J.** | *Diseases of India.* 2 vols. | (4) | 1828 |
| **Balme, H.** | *China and Modern Medicine* | | 1921 |
| **Bell, W. B.** | *The Sex Complex* | (7) | 1916 |
| **Bennett, R. R.** | *Materia Medica and Pharmacy* | 4th Ed. | 1921 |
| **Black, N.** | *Local Anæsthesia in Dental Surgery* | | 1921 |
| **Browne, A. R. I.** | *Medical Electricity for Students* | | 1921 |
| **Burnham, F. W. E.** | *Hæmocytes and Hæmic Infection* | (1) | 1913 |
| **Cook, W. G. H.** | *Insanity and Mental Deficiency* | | 1921 |
| **Cope, Z.** | *The Early Diagnosis of the Acute Abdomen* | | [1920] |
| **Fairbairn, J. S.** [Ed.] | *Encyclopædia of Midwifery and Diseases of Women* | | 1921 |
| **Flint, H. L.** | *The Heart: Old and New Views* | | 1921 |
| **Golgi, C.** | *Über den Bau des Nervensystem* | (6) | 1894 |
| **Harrison, L. W.** | *Venereal Disease in General Practice* | 3rd Ed. | [1921] |
| **Hart, B.** | *Modern Treatment of Mental and Nervous Disorders* | (4) | 1918 |
| **Lyle and D. de Souza, H.W.** | *Manual of Physiology* | [2nd Ed. | 1921] |
| **McCarrison, R.** | *Studies in Deficiency Disease* | | [1921] |
| **MacKenzie, Sir J.** | *Heart Disease and Pregnancy* | | 1921 |
| **Magnus, V.** | *Bidrag til hjernechirurgiens klinik* | | 1921 |
| **Monrad-Krohn, G. H.** | *Clinical Examination of the Nervous System* | | 1921 |
| **Morson, A. Abrahams and A. C.** | *A Guide to Urinary Diseases* | | 1921 |
| **Motherby, G.** | *Medical Dictionary* | (6) 5th Ed. | 1801 |
| **Moynihan, Sir B.** | *The Spleen and some of its Diseases* | | 1921 |
| **Orrin, H. C.** | *Aids to Operative Surgery* | | 1921 |
| **Plimmer, R. H. A.** | *Analyses and Energy Values of Foods* | | 1921 |
| **Quervain, F. de** | *Clinical Surgical Diagnosis* (Trans. by J. Snowman) | 3rd Eng. Ed. | 1921 |
| **Robertson, W. F.** | *Therapeutic Immunization* | | 1921 |
| **Sidis, B.** | *Psychopathic Diseases* | | 1921 |
| **Souza, H. W. Lyle and D. de.** | *Manual of Physiology* | [2nd Ed. | 1921] |
| **Talbot, E. S.** | *Developmental Pathology* | | 1921 |
| **Thomson, H. H.** | *Tuberculosis: Its Prevention and Home Treatment* | 2nd Ed. | [1921] |
| **Walters, F. R.** | *Domiciliary Treatment of Tuberculosis* | | 1921 |
| **Whitnall, S. E.** | *The Anatomy of the Human Orbit* | | [1921] |

### TRANSACTIONS, REPORTS, JOURNALS, &c.

| | | |
|---|---|---|
| American Association of Genito-Urinary Surgeons, Trans. of the | Vol. XIII. | 1920 |
| American Association of Obstetricians and Gynecologists, Trans. of the .. .. .. .. .. .. .. .. | Vol. XXXIII | 1921 |
| American Urological Association, Transactions of the .. .. .. | | 1921 |
| British Medical Journal, The .. .. .. | Vols. II. 1920 ; I. | 1921 |
| College of Physicians of Philadelphia, Transactions of | Vol. XLI. | 1919 |
| English Catalogue of Books for 1920, The .. .. .. .. .. | | 1921 |
| Lancet, The .. .. .. .. .. .. .. | Vols. II. 1920 ; I. | 1921 |
| Medical Annual, The .. .. .. .. .. .. .. .. .. .. | | 1921 |
| Memoirs of the Royal Society .. | (5) Vols. I,, IV., VI., IX. | 1738–41 |
| Quarterly Journal of Medicine, Index to Vols. I.–XII. .. | (2) | 1921 |

### Obituary.

UNIVERSITY OF BRISTOL.—Students of the University have recently passed the undermentioned examinations :—

M.S. LONDON.—*Surgery :* A. W. Adams.

M.B. LONDON.—*Intermediate Examination :* J. A. James, W. H. Royal.

M.B., CH.B. BRISTOL.—*First Examination :* Helen M. Aldwinkle, T. A. Barnabas, E. C. Bernard, B. J. Boulton, Muriel E. Drew, G. L. Feneley, W. G. Morris, L. B. Phillips, Dora L. Pratten, A. Vincent. *Botany only :* H. A. Jones.

*Second Examination (Part I.) :* H. C. Bentley, F. S. Dymond, F. J. Farr, Margaret P. Posthuma, E. May, E. S. Rogers. *Part II. :* P. G. Evans, P. C. Joscelyne, P. H. Knowles, K. F. Platt.

*Final Examination (Part I.) :* M. E. Golding.

D.P.H.—F. Campbell.

DIPLOMA IN DENTAL SURGERY.—*Final Examination :* K. G. Hyland, E. E. Lewis, J. C. Phillips.

B.D.S.—*Second Examination :* E. K. Tratman. *Third Examination :* H. N. Jones.

CONJOINT EXAMINING BOARD.—*Chemistry and Physics :* A. J. Penglaze. *Medicine :* M. Critchley, J. A. Pritchard, H. E. Reynolds, Mary Somers.

